# Characterization of Three *SEPALLATA-*Like MADS-Box Genes Associated With Floral Development in *Paphiopedilum henryanum* (Orchidaceae)

**DOI:** 10.3389/fpls.2022.916081

**Published:** 2022-05-26

**Authors:** Hao Cheng, Xiulan Xie, Maozhi Ren, Shuhua Yang, Xin Zhao, Nasser Mahna, Yi Liu, Yufeng Xu, Yukai Xiang, Hua Chai, Liang Zheng, Hong Ge, Ruidong Jia

**Affiliations:** ^1^Key Laboratory of Biology and Genetic Improvement of Flower Crops (North China), Ministry of Agriculture and Rural Affairs, Institute of Vegetables and Flowers, Chinese Academy of Agricultural Sciences, Beijing, China; ^2^National Agricultural Science & Technology Center, Institute of Urban Agriculture, Chinese Academy of Agricultural Sciences, Chengdu, China; ^3^Department of Horticultural Sciences, Faculty of Agriculture, University of Tabriz, Tabriz, Iran; ^4^Department of High-Performance Computing, National Supercomputing Center in Chengdu, Chengdu, China

**Keywords:** expression analysis, flower development, gene cloning, *Paphiopedilum*, *SEPALLATA-*like MADS-box genes

## Abstract

*Paphiopedilum* (Orchidaceae) is one of the world’s most popular orchids that is found in tropical and subtropical forests and has an enormous ornamental value. *SEPALLATA-*like (*SEP-*like) MADS-box genes are responsible for floral organ specification. In this study, three *SEP-*like MADS-box genes, *PhSEP1*, *PhSEP2*, and *PhSEP3*, were identified in *Paphiopedilum henryanum*. These genes were 732–916 bp, with conserved SEPI and SEPII motifs. Phylogenetic analysis revealed that *PhSEP* genes were evolutionarily closer to the core eudicot *SEP3* lineage, whereas none of them belonged to core eudicot *SEP1/2/4* clades. *PhSEP* genes displayed non-ubiquitous expression, which was detectable across all floral organs at all developmental stages of the flower buds. Furthermore, subcellular localization experiments revealed the localization of PhSEP proteins in the nucleus. Yeast two-hybrid assays revealed no self-activation of PhSEPs. The protein–protein interactions revealed that PhSEPs possibly interact with B-class DEFICIENS-like and E-class MADS-box proteins. Our study suggests that the three *SEP-*like genes may play key roles in flower development in *P. henryanum*, which will improve our understanding of the roles of the *SEP-*like MADS-box gene family and provide crucial insights into the mechanisms underlying floral development in orchids.

## Introduction

*Paphiopedilum* Pfitzer (Orchidaceae), commonly known as “slipper orchid,” is one of the world’s most popular orchids in the Orchidaceae family, owing to its remarkable diversity in terms of the shape, size, and color of flowers (Ng and Mohd Saleh, 2011; Zeng et al., 2013; Guo et al., 2021). This orchid can be mainly found in tropical and subtropical forests extending from Asia to the Pacific Islands. More than 18 species are widely distributed across Southwest China (Guan et al., 2011). *Paphiopedilum henryanum*, a species threatened with extinction, mainly occurs in the crevices of shady cliffs or rocks and well-drained habitats of the mountains along the Sino-Vietnamese border (Xu et al., 2018). The perianth of the *Paphiopedilum* flower consists of two petal-like sepals (whorl I), two lateral petals, and a highly diversified lip (whorl II). The inner fertile organ is adapted to represent gynostemium (whorl III; Pi et al., 2009). The reproductive organ of this ornamental plant is highly diversified and thus may serve as models for studying the molecular development of flowers in monocots.

Flower formation is known to be controlled by different regulatory genes, including several MADS-box family members (Ma et al., 2019). These MADS-box genes can be divided into two lineages, Type I and Type II, originating from a single-gene duplication that occurred before the divergence of plants and animals (Alvarez-Buylla et al., 2000). MADS-box proteins contain a highly conserved motif of 55–60 amino acids known as the MADS domain, which is essential for DNA-binding activity (De Bodt et al., 2003). As important transcriptional factors, MADS-box genes participate in various plant developmental processes, including the regulation of floral organ identity, inflorescence meristem identity, fruit ripening, and several other processes (Goto and Meyerowitz, 1994; Mandel and Yanofsky, 1995; Liljegren et al., 2000; Guo et al., 2017).

The developmental pathways for determining floral organ identity have been well-studied in several eudicot model species, such as *Arabidopsis thaliana* and *Antirrhinum majus* (Schwarz-Sommer et al., 1990; Coen and Meyerowitz, 1991). The ABCDE model of floral development was established as unifying paradigm and underlying principle of flower development and evolution. This model comprises five major classes of homeotic genes: A, B, C, D, and E. Except *APETALA2* (*AP2*), all of these genes belong to MADS-box genes (Theißen, 2001; Mondragõn-Palomino and Theißen, 2011). According to this model, the expression of A- and E-class genes leads to the development of sepals; the expression of A-, B-, and E-class genes give rise to petals; the expression of B-, C-, and E-class genes in the meristematic regions allows the development of stamens; carpels are formed when the C- and E-class genes are expressed; and ovules develop when the D- and E-class genes are expressed (Ditta et al., 2004; Theissen and Melzer, 2007; Pu and Xu, 2021).

*SEPALLATA* (*SEP*) are E-class MADS-box genes that act as important mediators of the higher-order complex and participate in various aspects of plant development together with B-, C-, and D-class MADS-box genes (Becker and Theißen, 2003; Immink et al., 2009; Pu et al., 2020). *SEP* genes have undergone two gene duplications during their evolution; the first duplication preceded the origin of the extant angiosperms, resulting in two clades, *AGL2/3/4* (*SEP1/2*) and *AGL9* (*SEP3*). Subsequent duplications have occurred independently within these clades after the divergence of eudicots and monocots (Shan et al., 2009). As for eudicots, *SEP* genes have been reported in tomatoes (*Solanum lycopersicum*), petunias (*Petunia hybrida*), and orchids (Ferrario et al., 2003; Uimari et al., 2004). Moreover, members of the SEP family have been identified in monocots, such as maize and rice (Becker and Theißen, 2003; Cui et al., 2010). In *Arabidopsis*, four *SEP* genes (*AtSEP1*, *AtSEP2*, *AtSEP3*, and *AtSEP4*) play a role in the development of all floral whorl and meristem determinacy (Ditta et al., 2004).

In orchids, the function of some MADS-box genes has been reported, and a specific model was established (Mondragón-Palomino, 2013). According to the “Homeotic Orchid Tepal” (HOT) model, the B-class genes in combination with genes of other classes, such as the E-class genes, regulate the complexity of sepal, petal, and lip identity (Pan et al., 2011). However, few *SEP-*like genes have been identified in orchid species, such as *AdOM1* in Aranda, *DcSEP1* in *Dendrobium crumenatum*, *DOMADS1* and *DOMADS3* in *Dendrobium grex Madame* Thong-IN, and *PeSEP1/2/3/4* in *Phalaenopsis equestris* (Lu et al., 1993; Xu et al., 2006; Pan et al., 2014). In Dendrobium, DcOSEP1/DcOPI/DcOAP3A or DcOAP3B (SEP-like/PI-like/AP3-like) could form multimeric proteins (Hsu et al., 2015). Functional analysis showed that virus-induced silencing of *PeSEP3* in *P. equestris* could alter the epidermal identity of tepals and the contents of anthocyanin and chlorophyll, causing tepals to become leaf-like organs (Pan et al., 2014). Agreeing well with research from Phalaenopsis, defects of *CeSEP1/3*-clade genes of the Chinese orchid *Cymbidium ensifolium* contributed to the leaf-like flower phenotype in the mutant, indicating that SEP paralogs differed in their ability to regulate floral organ specificity (Wei et al., 2020). Interestingly, *CeSEP-2* is important for the development of a specialized lip in Cymbidium orchids, while its downregulation resulted in the formation of a peloric flower shape in *C. ensifolium* (Ai et al., 2021). Further study revealed that the E-class MADS-box protein PeMADS8 in *P*. *equestris* could also interact with the B_sister_ protein PeMADS28, and a higher-order protein complex formed by C-E-D-Bsister genes (PeMADS1-PeMADS8-PeMADS-PeMADS28) was likely to be associated with regulation of orchid ovule development (Shen et al., 2021). In *Habenaria radiata*, the *SEP-*like gene *HrSEP1* plays an important role in column, lip, and petal development. The mutation in this gene can cause the greenish flower phenotype of *H. radiata* (Mitoma and Kanno, 2018). In contrast with the four genes found in other orchids, only two SEP transcripts were expressed in the inflorescence of *Orchis italica*, and both genes were detectable in all floral organs, which was consistent with the expression pattern in all the floral whorls of class E genes involved in the formation of all the organs of the flower (Valoroso et al., 2019). Moreover, *SEP-*like genes were also involved in orchid fruit development. In *Erycina pusilla*, the *SEP-like* genes *EpMADS8* and − *9* were expressed throughout fruit development, and protein–protein interaction studies revealed that MADS domain complexes comprised of SEP, FRUITFULL (FUL), and AGAMOUS (AG)/SHATTERPROOF (SHP) orthologs can also be formed in *E. pusilla* (Lin et al., 2016; Dirks-Mulder et al., 2019). To date, no *SEP* genes have been identified in *Paphiopedilum*. In addition, the flowers of *Paphiopedilum* contain a pocket-like lip and synsepal, distinguishing them from those of other orchids. Therefore, it is necessary to isolate and characterize the E-class genes of *Paphiopedilum* and address their developmental role in perianth identity.

In the present study, three *SEP-*like MADS-box genes were isolated from *P. henryanum*. The sequences of these genes and their encoded proteins were analyzed. Moreover, the expression patterns of *PhSEP* genes were explored using quantitative real-time PCR (qRT-PCR) performed on different tissues and organs and across various floral bud developmental stages. The subcellular localization and self-activation of the corresponding proteins were also investigated using the gene gun-mediated transformation and yeast two-hybrid system, respectively. In addition, the identification of PhSEPs’ binding sites was predicted by the DeepMind’s AlphaFold2 program. Thus, the present study aimed to establish a foundation for further studies by elaborating the molecular mechanisms of floral organ determination in slipper orchids.

## Materials and Methods

### Plant Material and Bacterial Strains

The *P. henryanum* plants used in this study were grown in a greenhouse at the Institute of Vegetables and Flowers, Chinese Academy of Agricultural Sciences. Floral buds representing developmental stages B1–B4 (stage B1: 2.0–3.0 mm in length; stage B2: 3.0–4.0 mm in length; stage B3: 4.0–5.0 mm in length; stage B4: 5.0–6.0 mm in length), various floral organs of mature flowers (sepal, petal, lip, ovary, gynostemium, and bract), scape, roots, and leaves were collected, immediately frozen in liquid nitrogen, and then stored at −80°C. Plasmid pEASY^®^-T3 (Takara, Japan) was used to clone the cDNA sequences, whereas the pBI221-EGFP and pGBKT7 (Clontech, United States) vectors were modified to clone overexpression constructs. *Escherichia coli* DH5α and *Saccharomyces cerevisiae* AH109 were used for transformation and self-activation, respectively.

### Cloning and Characterization of *PhSEP* Genes From *Paphiopedilum henryanum*

To identify, clone, and characterize the *SEP*-like genes of *P. henryanum*, total RNA was extracted from the harvested tissues using the RNAprep Pure Plant Kit (TIANGEN Biotech Co., Ltd., Beijing, China), according to the manufacturer’s instructions. Reverse transcription was carried out with 1.0 μg of each RNA sample using the FastQuant RT Kit (with gDNase; TIANGEN Biotech Co., Ltd., Beijing, China). Three *SEP-*like genes were identified from the transcriptome of *P. henryanum* (Accession nos. SRP131426 and PRJNA431671, available at the Sequence Read Archive (SRA) of the National Center for Biotechnology Information (NCBI) database). Based on the sequences retrieved, specific primer pairs (PhSEP1-F/PhSEP1-R, PhSEP2-F/PhSEP2-R, and PhSEP3-F/PhSEP3-R; Supplementary Table S1) were designed for cloning the coding sequences (CDS) of *PhSEP* genes. The amount of 1–2 μl of the synthesized cDNA (100 ng/μL) was used for PCR with primers and the high-fidelity Taq DNA polymerase (Ex Taq, TaKaRa Bio, Japan). The amplified products were evaluated by agarose gel electrophoresis and then cloned into the pEASY^®^-T3 vector. The recombinant clones were selected for amplification and identification. The nucleic acid sequences obtained were then compared with the homologous gene sequences retrieved from GenBank using Blastn. Open reading frame (ORF) search was performed using the online server getorf;^1^ the molecular weights and isoelectric points of the predicted proteins were analyzed using ProtParam,^2^ whereas their hydrophilicity was assessed using ProtScale;^3^ and the amino acid signal peptides and subcellular localization were predicted using SignalP3.0^4^ and PSORT,^5^ respectively.

### Multiple Sequence Alignment and Phylogenetic Analysis

*SEP*-like genes and *AP1/SQUA*-like genes were retrieved from previously published studies and other publicly available databases using BLAST searches (Pan et al., 2014). During the BLAST searches, multiple genes of the each subfamily from different lineages were used as queries. The following databases were used in the search: NCBI. Each of the databases was searched using TBLASTN. We obtained the sequences whose E-values were below le–5 and redundant sequences with identity of at least 95% were removed from our data set (Supplementary Table S2). Protein sequences were first aligned with MEGA11.^6^ Sequences of the alignment were ordered according to their phylogenetic placements in the preliminary tree, then, they were aligned manually using MEGA11and DNAMAN version 4.0 (Lynnon Biosoft Company; Kumar et al., 2016).

Phylogenetic analyses about *SEP3* were conducted using DNA alignments that included the conserved M-, I-, and K-domain regions and the C-terminal residues with higher than 12 quality scores. The quality score for each residue was estimated in CLUSTALX 2.1(Thompson et al., 1997). The PhyML software was used to construct ML tree with the most appropriate model, GTR + I + C, which was estimated by running MODELTEST version 3.06 and 1,000 bootstrap replicates (Posada and Crandall, 1998; Guindon and Gascuel, 2003). Bootstrapping was performed by resampling the data 1,000 times. Tree files were viewed using iTOL (Letunic and Bork, 2021).

### Gene Expression Analysis *via* qRT-PCR

To investigate the spatio-temporal expression patterns of the *SEP* genes, a quantitative reverse transcription PCR (qRT-PCR) was performed as described previously (Pan et al., 2014; Omondi et al., 2015; Shen et al., 2021) using tissues of roots, stems, leaves, floral organs of mature flowers, and developing floral buds at different stages. Gene-specific primers (qSEP1-F/qSEP1-R, qSEP2-F/qSEP2-R, and qSEP3-F/qSEP3-R) were designed within the non-conserved C-terminal region for each gene (Supplementary Table S1) using the Primer 5 software. The expected size of amplification products was 100–150 bp. TB Green^®^ Premix Ex Taq^™^ II (Tli RNaseH Plus; TaKaRa, Japan) was used for transcript quantification. The cycling program was as follows: an initial denaturation step at 95°C for 30 s, followed by 40 cycles of denaturation at 95°C (5 s), annealing at 60°C (30 s), and extension at 72°C (30 s). The relative mRNA abundance of the *SEP* genes and the reference gene, *Actin*, was analyzed using the 2^−ΔΔCt^ method (Livak and Schmittgen, 2001). Three independent biological replicates and three technical replicates were used for each experimental or control sample.

### Subcellular Localization of *PhSEP* Genes

To examine the subcellular localization of SEP genes, a gene gun was used for introducing DNA into onion (*Allium cepa*) inner epidermal cells as described previously by Wang et al. (2016) and modified by Li et al. (2020). To do this, the coding regions of *PhSEP* genes were amplified using KAPA HiFi^™^ HotStart DNA polymerase (KAPA Biosystems, United States), with the primers (DWSEP1-F/DWSEP1-R, DWSEP2-F/DWSEP2-R, DWSEP3-F/DWSEP3-R) listed in Supplementary Table S1. After purification, the PCR products were cloned downstream of the synthetic green fluorescent protein (EGFP) reporter gene in the pBI221-EGFP binary vector using the SE Seamless Cloning and Assembly Kit (Zomanbio, Beijing, China). The recombinant vector harboring the *SEP* fusion and the negative control (empty pBI221-EGFP vector) were used to transform living onion epidermal cells by biolistic bombardment using a Biolistc^®^ PDS-1000/He Particle Delivery System (Bio-Rad Laboratories, CA, United States) according to the manufacturer’s instructions (helium pressure, 9 MPa; Yu and Goh, 2000). Fluorescence was observed using a fluorescence microscope (BX53 Upright Microscope, Olympus, Tokyo, Japan).

### Yeast Assay and Protein–Protein Interactions Prediction

The yeast two-hybrid assay is a powerful and classic method of screening protein–protein interactions (Hu et al., 2021). To screen protein–protein interactions, a yeast two-hybrid assay was performed as described by Dirks-Mulder et al. (2019). The CDS of *SEP* genes were cloned in-frame downstream of the GAL4-binding domain of the pGBKT7 vector (Clontech, USA) after amplification with the primers (BDSEP1-F/BDSEP1-R, BDSEP2-F/BDSEP2-R, BDSEP3-F/BDSEP3-R) listed in Supplementary Table S1. The constructs were prepared using the SE Seamless Cloning and Assembly. The recombinant plasmids and the negative control (empty pGBKT7 vector) were used to transform *S. cerevisiae* AH109 competent cells according to the Yeast Protocols Handbook (Clontech, United States; De Folter and Immink, 2011). The cultures were serially diluted at a ratio of 1:10. Thereafter, 2 μl aliquots of the undiluted, 1:10, and 1:100 diluted cell cultures were spotted onto a non-selective medium, that is, the synthetic dropout medium without leucine and tryptophan (SD-LW), and selective media, including SD-LWH +3-AT (SD–leucine–tryptophan–histidine+5 mM 3-AT) and SD-LWHA (SD–leucine–tryptophan–histidine–adenine). The respective plates were incubated at 30°C for 7 days before being photographed. The self-activation of each protein was evaluated for its host status (Yeast Protocols Handbook; Clontech). AlphaFold2 (AF2) is a protein structure prediction model developed by DeepMind, which can predict the protein–protein complex structures and interaction accurately (Pozzati et al., 2021; Bryant et al., 2022). To predict the interaction ability of SEP proteins, a poly-glycine linker was added between each chain before running it as a single chain through the AlphaFold model (Humphreys et al., 2021; Tsaban et al., 2022). Molecular modeling was performed using the PyMOL molecular viewer for visualizing hydrogen bond interactions.

## Results

### Identification of *SEP* Genes From *Paphiopedilum henryanum* and Sequence Analysis

**Figure 1:**
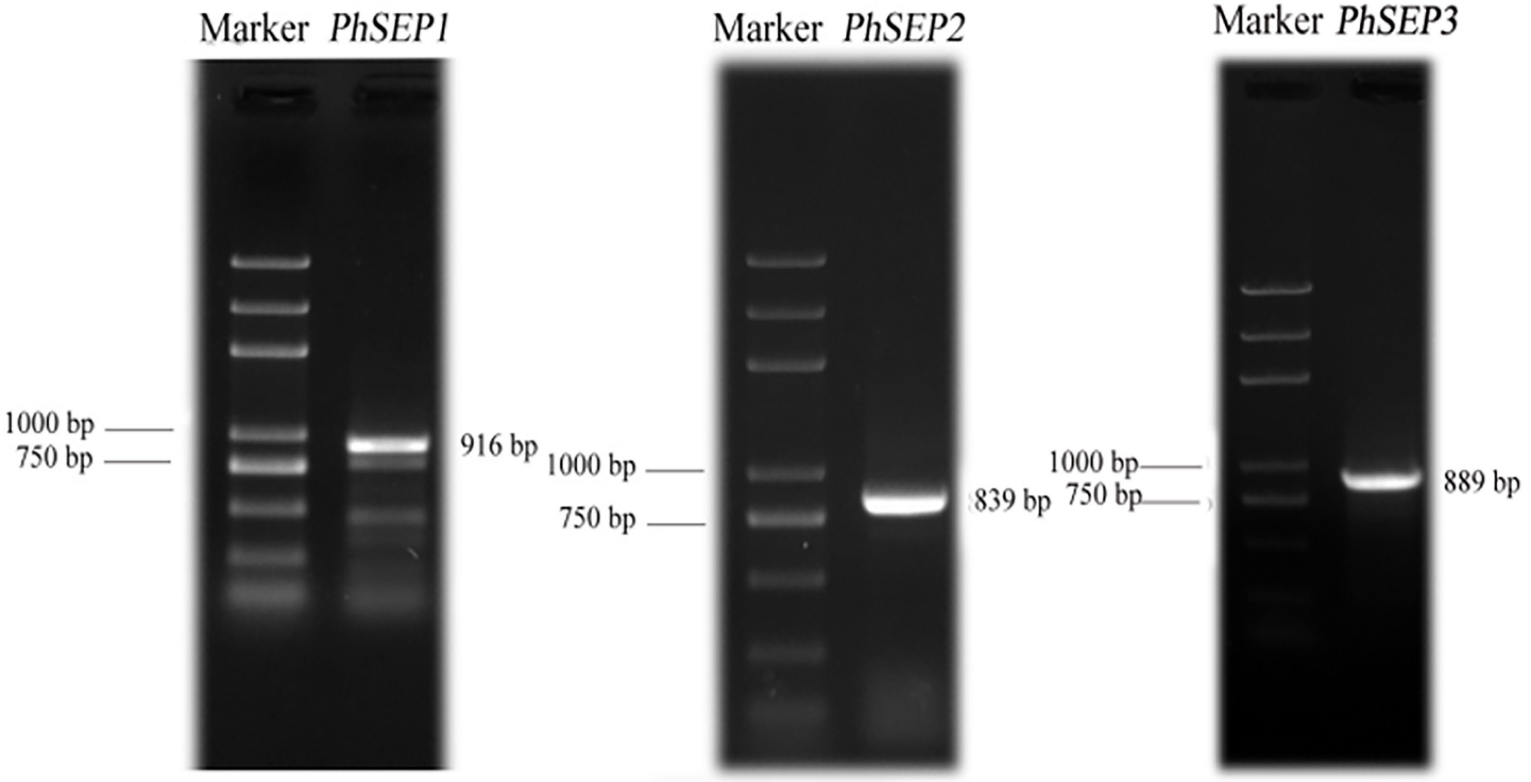
Amplification of three *PhSEP* genes from *Paphiopedilum henryanum.*

**Figure 2:**
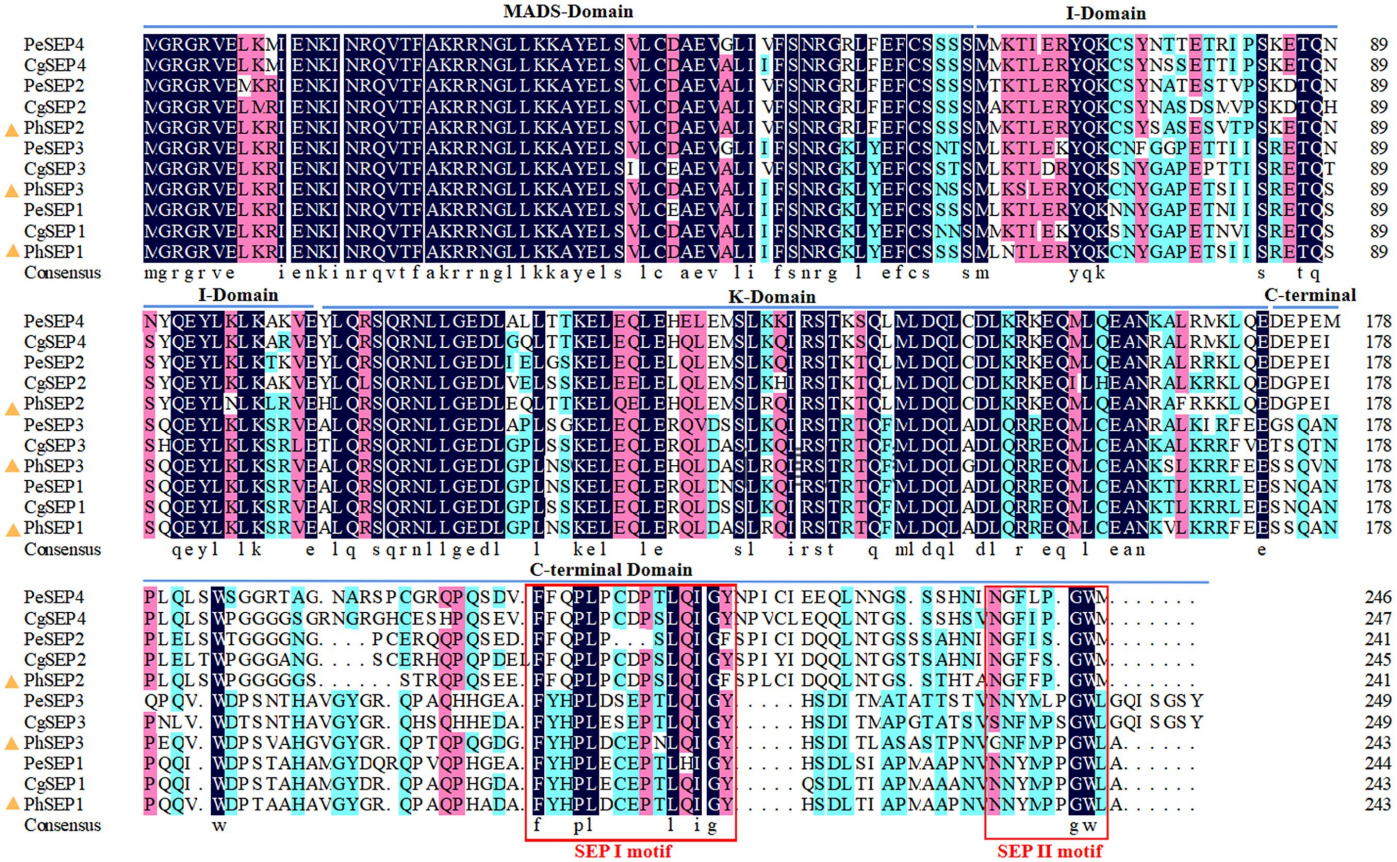
Amino acid sequence alignment of PhSEPs and closely related homologs in orchids using the MEGA11 and DNAMAN version 4.0. The PhSEP1/2/3 proteins of *P. henryanum* are highlighted by yellow triangles.

Three *SEP-*like genes were isolated from *P. henryanum* and named *PhSEP1*, *PhSEP2*, and *PhSEP3* (GenBank accession nos. MN274961, MN274962, and MN809620, respectively; Figure 1). *PhSEP1* was 916 bp in length and contained an ORF of 732 bp. *PhSEP2* was 839 bp in length and contained an ORF of 726 bp, whereas *PhSEP3* was 889 bp in length with a 732 bp ORF. *PhSEP1* shared 86% identity with its *P. equestris* homolog, *PeSEP1*. *PhSEP2* and *PhSEP3* independently showed 81% identity with *PeSEP2* and *PeSEP1*, respectively. The predicted proteins showed length of 241 (PhSEP2) and 243 (PhSEP1/3) amino acids and pI ranging between 8.71 (PhSEP2) and 8.94 (PhSEP1). Further bioinformatics analysis showed that these PhSEP proteins displayed a theoretical molecular mass of 28 kDa and harbored a nuclear localization signal. In addition, these proteins lacked transmembrane domains. Multiple sequence alignment with homologous SEP proteins from orchids (Figure 2) indicated that PhSEPs harbored a conserved MIK domain and divergent C-terminal domain with conserved SEP I and SEP II motifs, which are characteristic of E-class MADS-box proteins.

### Phylogenetic Analysis of *SEP*-Like Genes

**Figure 3:**
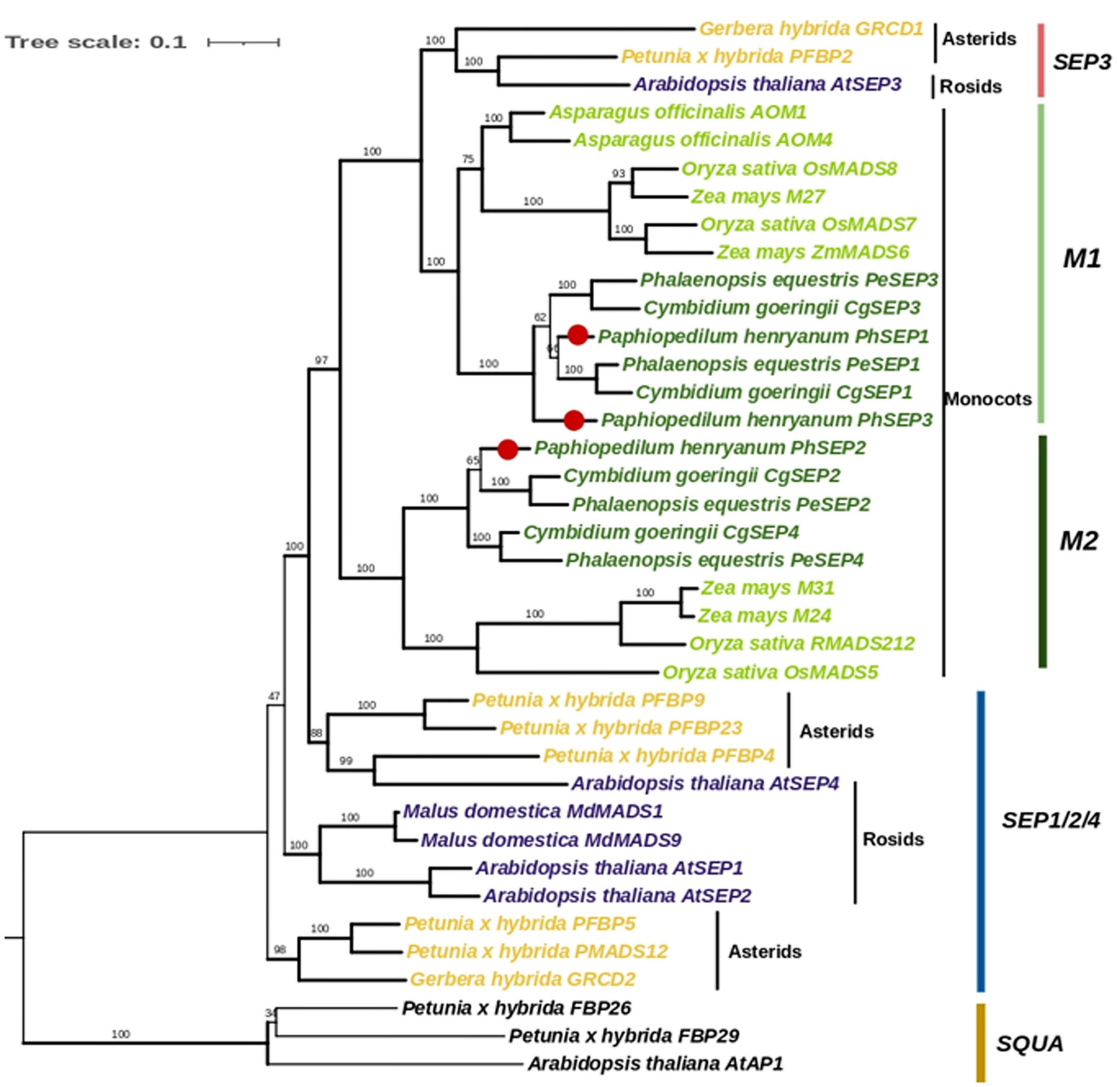
Phylogenetic analysis of *SEP*-like genes with *SQUA* genes as an outgroup. The topology of this tree was generated using PhyML. ML bootstrap support (MLBS) values are indicated on each branch. Thick branches indicate high support values with MLBS ≥ 70. PhSEPs are marked with red dots.

To determine the evolutionary relationships of the SEP subfamily within orchids and with other angiosperms, we constructed a phylogenetic tree using nucleic acid sequences. Totally, 35 *SEP* genes from orchids, other monocots, asterids, and rosids were obtained (Supplementary Table S2). We then performed phylogenetic analyses on the nucleotide sequences of these genes using Maximum Likelihood (ML), with 3 *AP1/SQUA*-like genes as the outgroups. The phylogenetic tree showed that *SEP* genes from monocots formed a well-supported single clade (Figure 3). They were divided into two major clades, *M1* and M2, with strong supporting values. The clade *M1* (including *PhSEP1* and *PhSEP3*) was clustered together with the *SEP3* genes of eudicots, leaving the clade *M2* (including *PhSEP2*) alone (well-supported), suggesting a duplicate event before the separation of monocots and eudicots. Monocots *SEPs* selected were not included in core eudicot *SEP1/2/4*, differently from previous study (Pan et al., 2014). A neighbor-joining based phylogenetic tree of 50 *SEP*-like genes showed that *PhSEP1* and *PhSEP3* were included in the *SEP3* clade while *PhSEP2* was grouped into the *SEP1/2/4* clade (Supplementary Figure S2).

### Expression Analysis of *PhSEP* Genes

**Figure 4:**
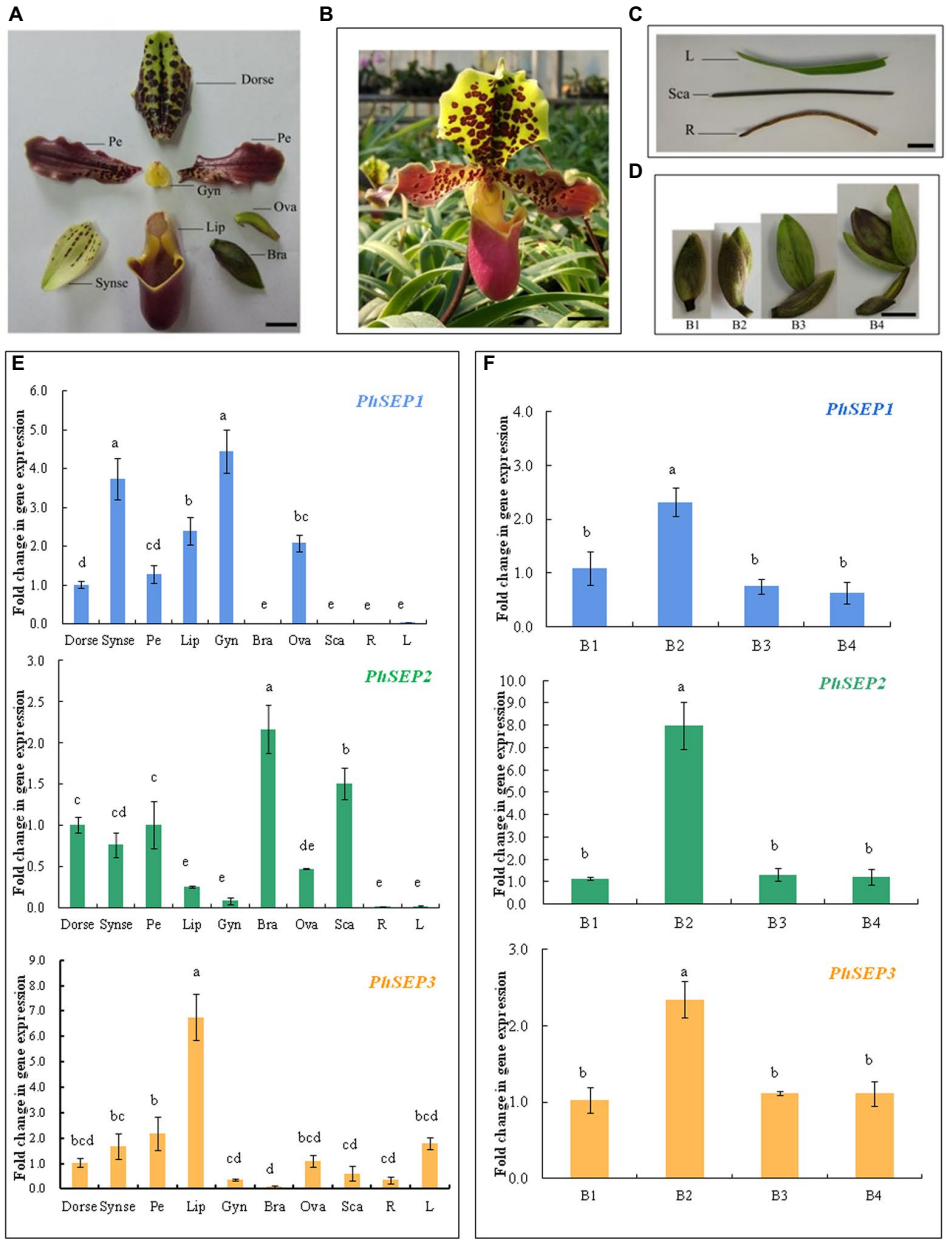
Transcript levels of PhSEP genes in different tissues and organs and at different developmental stages of floral buds of *P. henryanum*. **(A)** A flower dissected as follows: Dorse, dorsal; sepal; Synse, synsepal; Pe, petal; Lip; Gyn, gynostemium; Bra, bract; Ova, ovary. **(B)** Mature flowers. **(C)** Vegetative tissues dissected as follows: R, root; Sca, scape; L, leaf. **(D)** Floral buds at different developmental stages. **(E)** Relative expression patterns of PhSEP1, PhSEP2, and PhSEP3 in different tissues and organs. **(F)** Relative expression patterns of PhSEP1, PhSEP2, and PhSEP3 at four stages of floral development. Scale bars: 10 mm; The values are means of three replicates ± SE. Statistical analysis was performed using one-way ANOVA test (*p* < 0.05). Different letters represent significant difference. The expression level of gene in Dorse or at stage B1 was set to 1, and those of others were normalized to it.

qRT-PCR was performed to determine the spatio-temporal expression pattern of *PhSEP* genes across different tissues and organs of *P. henryanum* (Figures 4A–D). As shown in Figure 4E, the expression of *PhSEP1* was specific to reproductive tissue and was especially high in the gynostemium and synsepal. In contrast, its expression was negligible in vegetative tissues, including the scape, roots, and leaves. *PhSEP2* was expressed in all reproductive tissues and the scape. However, it displayed negligible expression in the roots and leaves. *PhSEP3* was predominantly expressed in the petals, dorsal sepals, ovaries, and especially lips. We examined the temporal expression pattern of *PhSEP* genes in floral buds at four developmental stages (Figures 4D,F). Abundant *PhSEP* transcripts were found throughout floral development, whereas stage B2 showed the highest transcript accumulation. Overall, the expression patterns of *PhSEP* genes indicated that *PhSEP* genes play multiple roles in the flower development of *P. henryanum*.

### Subcellular Localization of PhSEP Proteins

**Figure 5:**
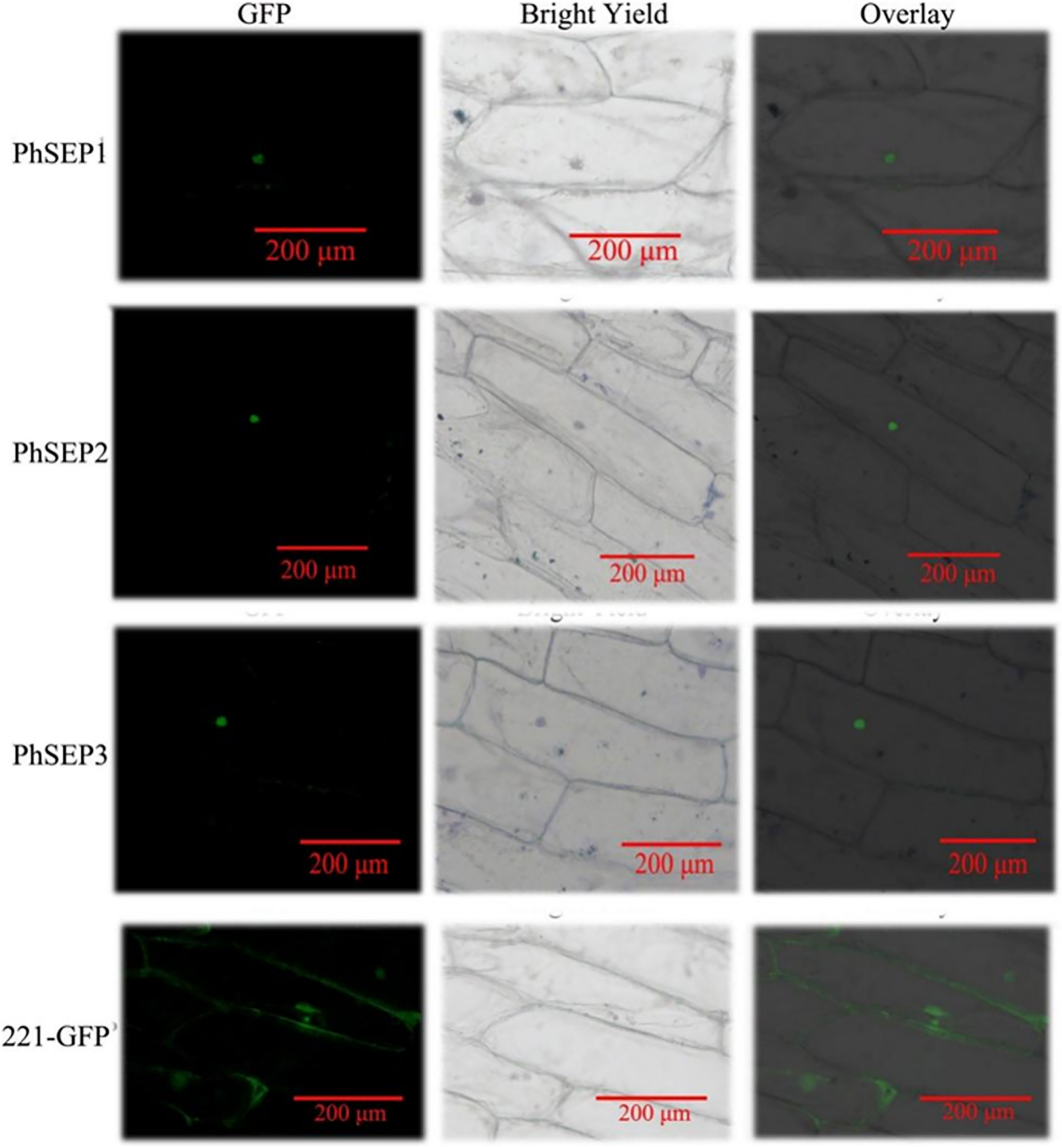
Subcellular localization of the putative PhSEPs in onion (*Allium cepa*) inner epidermal cells. The onion epidermal cells transiently expressing PhSEP1–GFP, PhSEP2–GFP, and PhSEP1–GFP fusion proteins and the EGFP control were visualized through fluorescence microscopy. In each horizontal panel, the extreme left represents GFP fluorescence, the middle image represents bright field, and the right image represents an overlay of the other two images.

To investigate the subcellular localization of PhSEP proteins, the EGFP–PhSEP fusion constructs and EGFP control were cloned in pBI221 under the regulatory control of the CaMV35S promoter. These constructs were transiently expressed in onion epidermal cells and analyzed by fluorescence microscopy. It was found that the PhSEP1–GFP, PhSEP2–GFP, and PhSEP1–GFP fusion proteins harboring nuclear localization signals were targeted to the nucleus, whereas the control GFP protein was localized in the cytosol and nucleus (Figure 5). These results indicated that PhSEPs are in fact nuclear proteins, in agreement with their role as transcription factors (TFs).

### Self-Activation Detection and Protein–Protein Interaction Prediction

**Figure 6:**
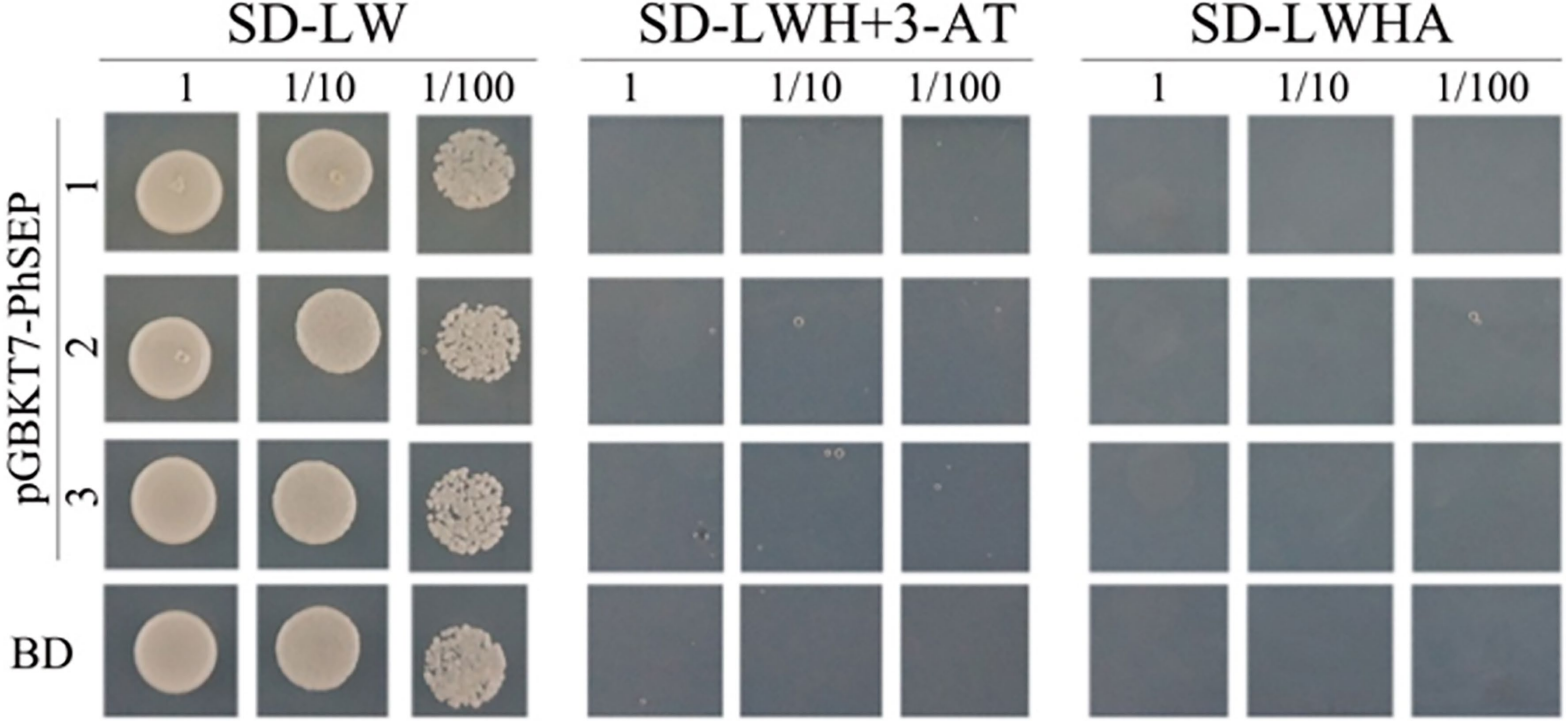
Evaluation of the self-activation ability of PhSEPs in budding yeast. The yeast cells of strain AH109 harboring the indicated plasmids were grown on non-selective (SD-LW) or selective (SD-LWH + 3-AT and SD-LWHA) media. Decreasing cell densities represent the 10-fold dilution series. BD in the last row represents an empty GAL4 DNA-binding domain containing a vector.

To investigate whether PhSEPs could be self-activated, we analyzed the ability of PhSEPs to activate the reporter genes *LacZ*, *TRP1*, *LEU1*, and *ADE2* in budding yeast. To this end, the CDS of the *PhSEP* genes were fused to the *GAL4* DNA-binding domain, and their ability to activate transcription from the *GAL4* upstream activation sequence (UAS) was assessed in terms of yeast growth. The yeast cells contained individual *PhSEP* plasmids and the control plasmid were sustained well on the non-selective SD-LW medium, whereas growth was absent on the selective SD-LWH + 3-AT or SD-LWHA medium, suggesting that no self-activation activity of PhSEPs was confirmed in yeast cells (Figure 6). In addition, the change in the color of the colonies from pink to red further indicated that the *ADE2* reporter gene was not expressed.

**Figure 7:**
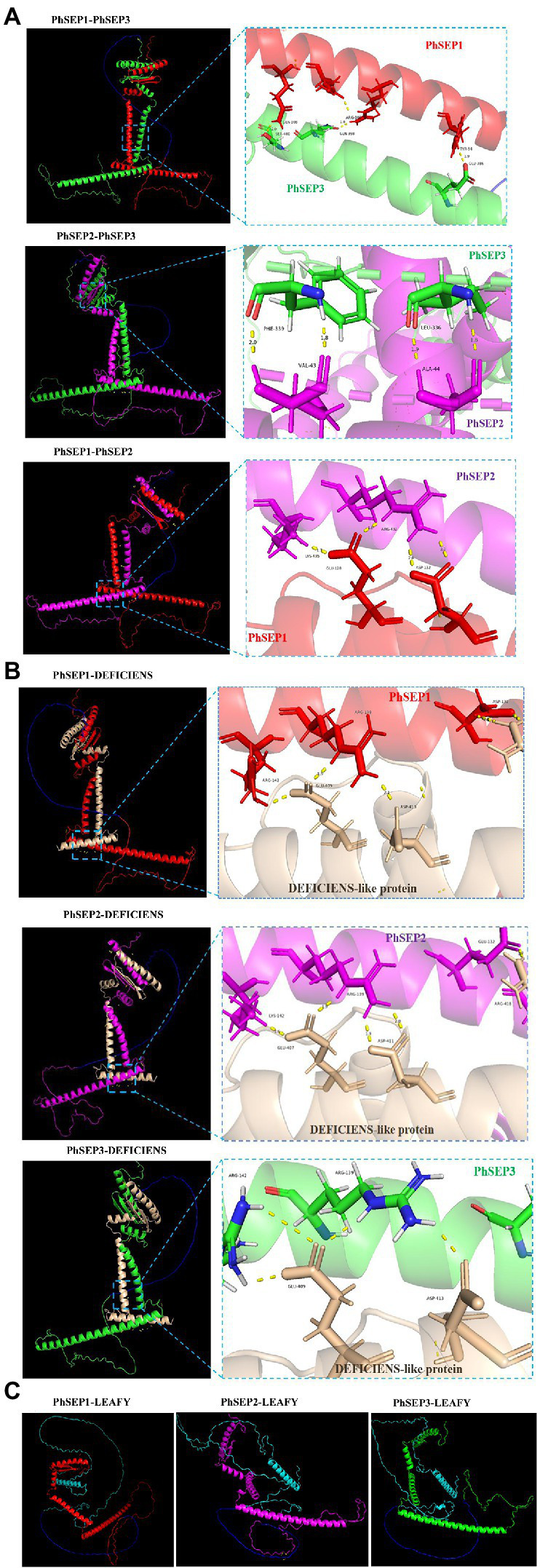
The prediction of protein–protein interactions by DeepMind’s AlphaFold2 program. **(A)** The prediction of PhSEPs-PhSEPs interactions. **(B)** The prediction of PhSEPs-DEFICIENS interactions. **(C)** The prediction of PhSEPs-LEAFY interactions. The red chain represents the structure of the PhSEP1 protein. The magenta chain represents the structure of the PhSEP2 protein. The green chain represents the structure of the PhSEP3 protein. The wheat chain represents the structure of the DEFICIENS-like protein. The cyan chain represents the structure of the LEAFY protein. The blue chain represents the linker. The yellow stick represents the hydrogen bonds between any of the two proteins. In **(A,B)** each horizontal panel, the left image represents the predicted structure of the protein complex, and the right image represents the predicted protein interaction sites.

The neural network AlphaFold2 developed by the artificial intelligence company DeepMind was trained using multiple sequence alignments (MSA) and experimental protein structures deposited before April 30, 2018. It could be used to predict the protein structure at the atomic level with high accuracy. Besides, the protein–protein interactions could be also predicted by AlphaFold2 (Tunyasuvunakool et al., 2021; Hegeds et al., 2022). In this study, three SEP proteins, DEFICIENS-like protein (AKC93996.1) and flower meristem identity protein LEAFY (AKC94104.1) in *P. henryanum* were selected for the prediction of the protein–protein interactions (Figure 7). The structural models of the protein complexes revealed that PhSEP1-PhSEP2, PhSEP1-PhSEP3, and PhSEP2-PhSEP3 interaction were likely to have existed (Figure 7A). Besides, we also demonstrated that PhSEPs might interact with DEFICIENS-like proteins (Figure 7B). In Figure 7C, the structure of PhSEPs-LEAFY complexes was dispersed, and no hydrogen bonds were observed between the proteins, which indicates a potential lack of protein–protein interactions between PhSEPs and the central floral development protein LEAFY.

## Discussion

With more than 25,000 species, orchids are the second-largest plant family (Stokstad, 2015; Andriamihaja et al., 2021). They have a unique zygomorphic floral structure, including three sepals, two petals, and a highly diversified lip (Rudall and Bateman, 2002). The highly specialized and diverse morphology of flowers in orchids makes them excellent models for examining the complex network of regulatory genes involved in floral morphogenesis (Pan et al., 2011). Recently, *SEP*-like genes have been identified and characterized in a wide range of eudicots and monocots, including Arabidopsis, rice, and orchid (Pu et al., 2020; Adal et al., 2021; Zhu et al., 2022). Numerous reports have shown that these genes are instrumental in the floral evolution of diverse plants and play fundamental roles in floral organ fate determination during development by interacting with other MADS-box gene products, such as those from A-, B-, and C-class genes (Malcomber and Kellogg, 2005; Mitoma and Kanno, 2018; Qi et al., 2020; Pu and Xu, 2021).

In the present study, three *SEP*-like genes, *PhSEP1*, *PhSEP2*, and *PhSEP3*, were cloned from *Paphiopedilum* orchid. Sequence and phylogenetic analysis revealed that *PhSEP* genes from *Paphiopedilum* were highly conserved. In addition, the predicted amino acid sequences of PhSEPs showed a high degree of identity with homologous proteins from *P. equestris*, *Cymbidium goeringii*, and *A. thaliana* (Ditta et al., 2004; Pan et al., 2014; Yang et al., 2021). In general, a similar primary structure of proteins represents a relatively close evolutionary relationship, analogous structure, and identical functions (Ma et al., 2019). Furthermore, the conservation of SEP I and SEP II motifs in the highly variable C-terminus of PhSEPs supported their characterization as E-class floral meristem identity genes and suggested a similar functionality to their orthologs in other plants. Duplication events are common in MIKC-type MADS-box TFs and many MIKC-type homoeologs are functionally important and not redundant (Shan et al., 2009; Schilling et al., 2020). Consistent with this fact, several duplication events have been reported in the evolutionary lineages of *SEP* genes in both eudicots and monocots, resulting in four *SEP* members in *Arabidopsis*, *Cymbidium*, and *Phalaenopsis* (Ditta et al., 2004; Chang et al., 2009; Mondragón-Palomino, 2013; Pan et al., 2014). Extensive duplication of MADS-box genes and the resulting subfunctional and expressional differentiation were associated with regulation of species-specific flower traits, such as floral patterning, seasonal flowering, and ecological adaption (Yang et al., 2021). Phylogenetic analysis showed that the three *PhSEP* genes were sorted into two diversified clades (*M1* and the *M2* clade in monocots), in consistent with the findings of previous studies reporting the phylogeny of *SEP*-like genes from *P. equestris* and *C. goeringii*. According to the phylogenetic tree, three *PhSEP* genes from *Paphiopedilum* were clustered together with *SEP1/2/3* genes from other *P. equestris* and *C. goeringii*, respectively (Pan et al., 2014; Yang et al., 2021), so it is quite possible to clone *PhSEP4*, the orthologous orchid *SEP4* gene. Hence, according to our analysis result, the monocots *SEPs* did not belong to *SEP1/2/4* from eudicots, differently from previous study (Pan et al., 2014). We speculated that there might be more unidentified *SEP* genes in orchids if they were not lost in plants evolution. The frequent duplication of *SEP* genes might be one of the main cause of diversity of flower structure in angiosperms.

*SEP-like* genes encode MADS transcription factors required for the formation of all the organs of the flower and for the determinacy of the floral meristems (Valoroso et al., 2019; Pu and Xu, 2021). In this research, the *PhSEP* genes displayed differential spatial expression patterns in vegetative and reproductive tissues of *P. henryanum*. *PhSEP* genes were collectively expressed in all flower organs, as observed earlier in other plants (Xu et al., 2006; Pan et al., 2014; Adal et al., 2021). The expression levels of *PhSEP* genes in roots and leaves were negligible. These findings suggest that the *SEP-*like genes in orchids are involved in the specification of floral organ identity. Interestingly, *PhSEP1* displayed expression patterns complementary to those of *PhSEP2*. While high expression levels of *PhSEP1* were noted in the synsepal, lip, gynostemium, and ovary, *SEP2* showed high expression levels in bracts and scapes. This result agreed well from research from *C. ensifolium*. The expression of the *CeSEP1/3*-clade genes TDN29274 and TDN28990, the orthologs of *PeSEP1* and *PeSEP3*, respectively, was obviously reduced. However, the other two *CeSEP* genes showed equal or slightly higher expression in the leaf-like flower mutant of *C. ensifolium* (Wei et al., 2020). Similarly, the expression levels of *BroaSEP1/2/3* genes from *Brassica oleracea* were very different at different developmental stages, also in the wild type, mutant flower with increased petals, and mutant flower with decreased petals, which indicated that different patterns of gene expression may cause the flowers to increase or decrease the petal number (Xiang et al., 2020). Moreover, the expression patterns of *PhSEP2* were reminiscent of those of its orthologs *CgSEP2* and *PeSEP2*, which also display substantial expression in sepals and petals and are minimally expressed in the lip (Pan et al., 2014; Yang et al., 2021). The non-overlapping expression profiles of the *PhSEP* genes indicate possible functional divergence. One possible reason for this divergence may be problems caused by changes in the exon–intron structure of the SEP subfamily (Yu et al., 2016; Schilling et al., 2020). *SEP3* and its orthologs, such as *FBP2* (petunia), *TM5* (tomato), *WSEP* (wheat), and *EScaAGL9* from the basal eudicot California poppy (*Eschscholzia californica*), are only expressed in the inner three whorls of the flower (Angenent et al., 1992, 1994; Pelaz et al., 2000). In contrast, the *PhSEP3* transcripts were detected in all floral organ whorls, especially in lip, indicating that *PhSEP3* might be the key gene associated with the lip; thus, the mRNA expression pattern of *PhSEP3* was slightly different from that of its aforementioned orthologs. This finding may be attributed to the remarkable similarity between the sepals and petals of the flowers of *P. henryanum*, implying that the genes that control petal formation in slipper orchids might be similarly expressed in sepals (Chang et al., 2009).

TFs, a major driver in evolution and in domestication, can facilitate or obstruct the access of RNA polymerases to the DNA template in association with other transcriptional regulators, including chromatin-remodeling/modifying proteins (Udvardi et al., 2007; Martínez-Ainsworth and Tenaillon, 2016). MADS-box genes constitute one of the largest families of plant TFs (Riechmann et al., 2000). The *SEP* genes, which are E-class MADS-box TFs, play vital roles in various aspects of plant growth and development (Qi et al., 2020). In this study, three PhSEPs were located in the nucleus, indicating the possible involvement of these TFs proteins in the regulation of the expression of downstream genes associated with floral development. Recent yeast two-hybrid experiments demonstrated that SEP proteins have conserved interactions with other MADS-box proteins of the SQUA, DEF/GLO, and AG subfamilies (Zahn et al., 2005). Moreover, SEP-like proteins can interact with FUL-like proteins during fruit patterning of *E. pusilla* (Dirks-Mulder et al., 2019). Consistent with this role, PhSEPs were incapable of self-activation and the prediction of protein–protein interactions by AlphaFold2 showed that PhSEPs might interact with PhSEPs and B-class DEFICIENS-like proteins. Furthermore, the central floral development protein LEAFY is necessary in triggering flower formation on inflorescences, while the SEP family can reprogram cauline leaves into the floral organs. LEAFY promotes floral fate through upregulation of the floral commitment factor A-class APETALA1 (AP1; Jin et al., 2021). We found that LEAFY might not interact with E-class SEP proteins in *P. henryanum*. Previous studies reported that the greenish flower phenotype of *Habenaria radiata* (Orchidaceae) is caused by a mutation in the *SEP*-like MADS-box gene *HrSEP-1* (Mitoma and Kanno, 2018). In *Lavandula angustifolia*, the expression of lavender *SEP*-like genes promote early flowering and alter leaf morphology in *A. thaliana* (Adal et al., 2021). In *Apostasia shenzhenica*, the adaxial petal does not differentiate into a specialized lip due to the loss of class B-AP3 and E-class genes (Zhang et al., 2017). Although there was no loss of B and E clade genes in *C. ensifolium*, transcriptomic analysis showed that the upregulation of *CeSEP-2* is necessary for the development of a specialized lip in *Cymbidium* orchids, while its downregulation results in the formation of a peloric flower shape (Ai et al., 2021). In addition, *CsSEP4* was originally found to positively regulate gynostemium development in *Cymbidium sinense*. The gene was ectopically expressed in the gynostemium of the wild-type flower and expended to all floral organs of a gynostemium-like perianth variant in *C. sinense*, and the *35S:CsSEP4 Arabidopsis* showed a severe flower phenotype whereas the *35S:CsSEP3* had an abnormal stamen and ovule (Yang et al., 2021). These results reveals that *SEP*-like genes are associated with the development of flower organs, especially for the lip. Thus, *PhSEP1/2/3* genes might have a similar function in floral organ identity. As in planta transformation of *Paphiopedilum maudiae* by agrobacterium-mediated ovary-injection was established, *FT* (*Flowering Locus T*) functional genes of *P. maudiae* were transformed into Paphiopedilum to elucidate their role during floral bud development and shorten the juvenile phase (Luo et al., 2020). This may be a useful way for the transformation of *PhSEP* genes in *P. henryanum*. There are differences in expression patterns and functional differentiation among paralogous genes or even among orthologous genes in closely related species (Morel et al., 2019; Yang et al., 2021). Functional differentiation might exist within the three *SEP*-like genes of *P. henryanum*.

The molecular basis of orchid flower development is accomplished through a specific regulatory program, and *SEP*-like genes enrich the molecular program underpinning the orchid perianth development, resulting in the expansion of the original “orchid code” in an even more complex gene regulatory network (Lucibelli et al., 2021). According to the quartet model, E-class genes are essential for the formation of quaternary complexes (Mitoma and Kanno, 2018). We suspected that *PhSEPs* genes may act as the “glue” for MADS-box transcription factor complex formation to regulate perianth formation in *P. henryanum*. In recent years, the genomes of some orchids including *Apostasia shenzhenica*, *Phalaenopsis aphrodite*, *Vanilla planifolia*, *C. goeringii*, *Dendrobium chrysotoxum*, *Platanthera zijinensis*, and *Platanthera guangdongensis* were published (Zhang et al., 2017, 2021; Chao et al., 2018; Hasing et al., 2020; Sun et al., 2021; Li et al., 2022). By contrast, the occurrence of whole-genome duplication in *Paphiopedilum* results in a genome that is very large and complex Besides, A-, B-, C-, D-class MADS-box genes in *P. henryanum* have not been systematically identified, which restricts the analysis of the protein–protein interaction network. Moreover, the tissue culture and genetic transformation systems are not applied widely in *Paphiopedilum* industry, and the mutant of this flower is difficult to be obtained and preserved. More functional data are required to validate our orchid flower regulatory model, such as breeding *PhSEP* genes-overexpressing and gene-silenced mutants by virus-induced gene silencing and transgenic technology, protein–protein interaction validation by yeast two-hybrid system and bimolecular fluorescence complementation, and downstream target genes detection by chromatin immunoprecipitation, electrophoretic mobility shift assay, and dual-luciferase. The biological function of *PhSEP* genes still needs to be evaluated in the *Paphiopedilum* or model plant *Arabidopsis*. Further research is required to explore the mechanisms underlying floral development.

## Conclusion

In this study, three *SEP-*like MADS-box genes in slipper orchids were identified for the first time, and the characteristics and expression patterns of the gene and protein sequences were systematically analyzed. All three homologs were structurally conserved and were characterized as E-class MADS-box transcription factors. Phylogenetic analysis revealed that *PhSEP1*, *PhSEP2*, and *PhSEP3* were evolutionarily closer to the core eudicot *SEP3* lineage, whereas none of them belonged to core eudicot *SEP1/2/4* clade. *PhSEP* genes were expressed during flower development and exhibited non-ubiquitous expression patterns. All SEP proteins were localized to the nucleus. Furthermore, we observed no self-activation of SEP proteins and the prediction of protein–protein interactions by AlphaFold2 revealed that SEP proteins might interact with SEP and DEFICIENS-like proteins. Consequently, these results illustrate three *SEP-*like MADS-box genes *PhSEP1*, *PhSEP2*, and *PhSEP3* might play a vital role in flower development in *P. henryanum*. Future research needs to be conducted to further elucidate the regulatory networks underlying the floral development and organ identity in the slipper orchid.

## Data Availability Statement

The original contributions presented in the study are included in the article/Supplementary Material, further inquiries can be directed to the corresponding authors.

## Author Contributions

RJ, HG, and MR: conceptualization and supervision. XX, SY, XZ, and YL: methodology. RJ, YXi, HC, and LZ: software. YXu and NM: validation. HC and XX: formal analysis and investigation. RJ, HG, SY, and XZ: resources. HC and YL: data curation. HC: writing—original draft preparation and visualization. HC, XX, NM, and YL: writing—review and editing. HG and RJ: project administration and funding acquisition. All authors contributed to the article and approved the submitted version.

## Funding

This work was financially supported by the National Natural Science Foundation of China (grant number 31301810), the Special Fund for Agro-scientific Research in the Public Interest (grant number 201203071), and the Agricultural Science and Technology Innovation Program of the Chinese Academy of Agricultural Sciences (grant number 34-IUA-02).

## Conflict of Interest

The authors declare that the research was conducted in the absence of any commercial or financial relationships that could be construed as a potential conflict of interest.

## Publisher’s Note

All claims expressed in this article are solely those of the authors and do not necessarily represent those of their affiliated organizations, or those of the publisher, the editors and the reviewers. Any product that may be evaluated in this article, or claim that may be made by its manufacturer, is not guaranteed or endorsed by the publisher.

## Footnotes

^1^ http://emboss.bioinformatics.nl/cgi-bin/emboss/getorf

^2^https://web.expasy.org/protparam/

^3^ https://web.expasy.org/protscale/

^4^ http://www.cbs.dtu.dk/services/SignalP-3.0/

^5^ https://www.genscript.com/psort.html

^6^ https://www.megasoftware.net/
