# Supplementary figures and images for: Characterization of Three SEPALLATA-Like MADS-Box Genes Associated With Floral Development in Paphiopedilum henryanum (Orchidaceae)
Source: Front Plant Sci. 2022 May 26;13:916081. doi: 10.3389/fpls.2022.916081 (PMC9178235; doi:10.3389/fpls.2022.916081)

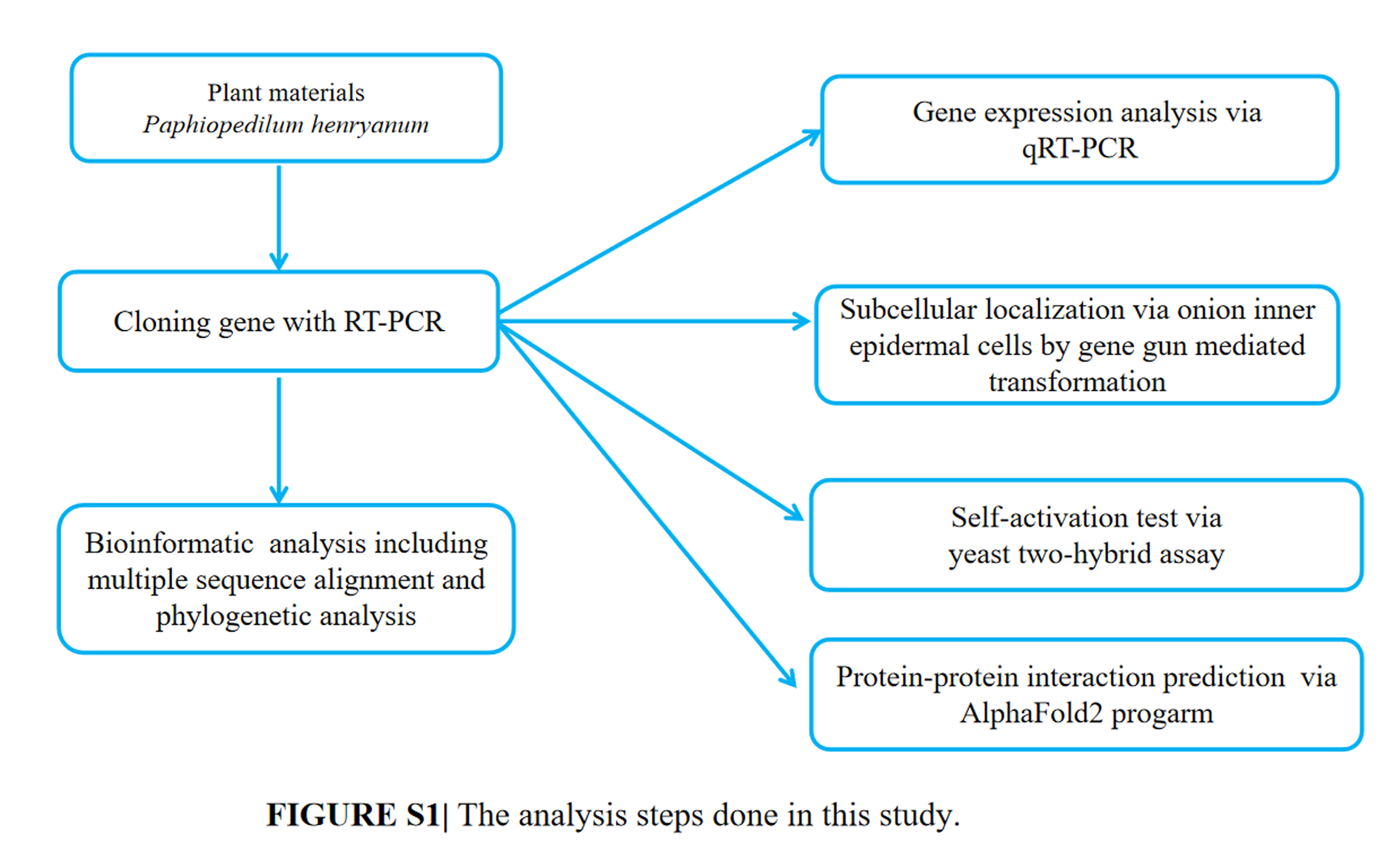

Supplement: Supplementary file 3 [file Image_1.TIF]

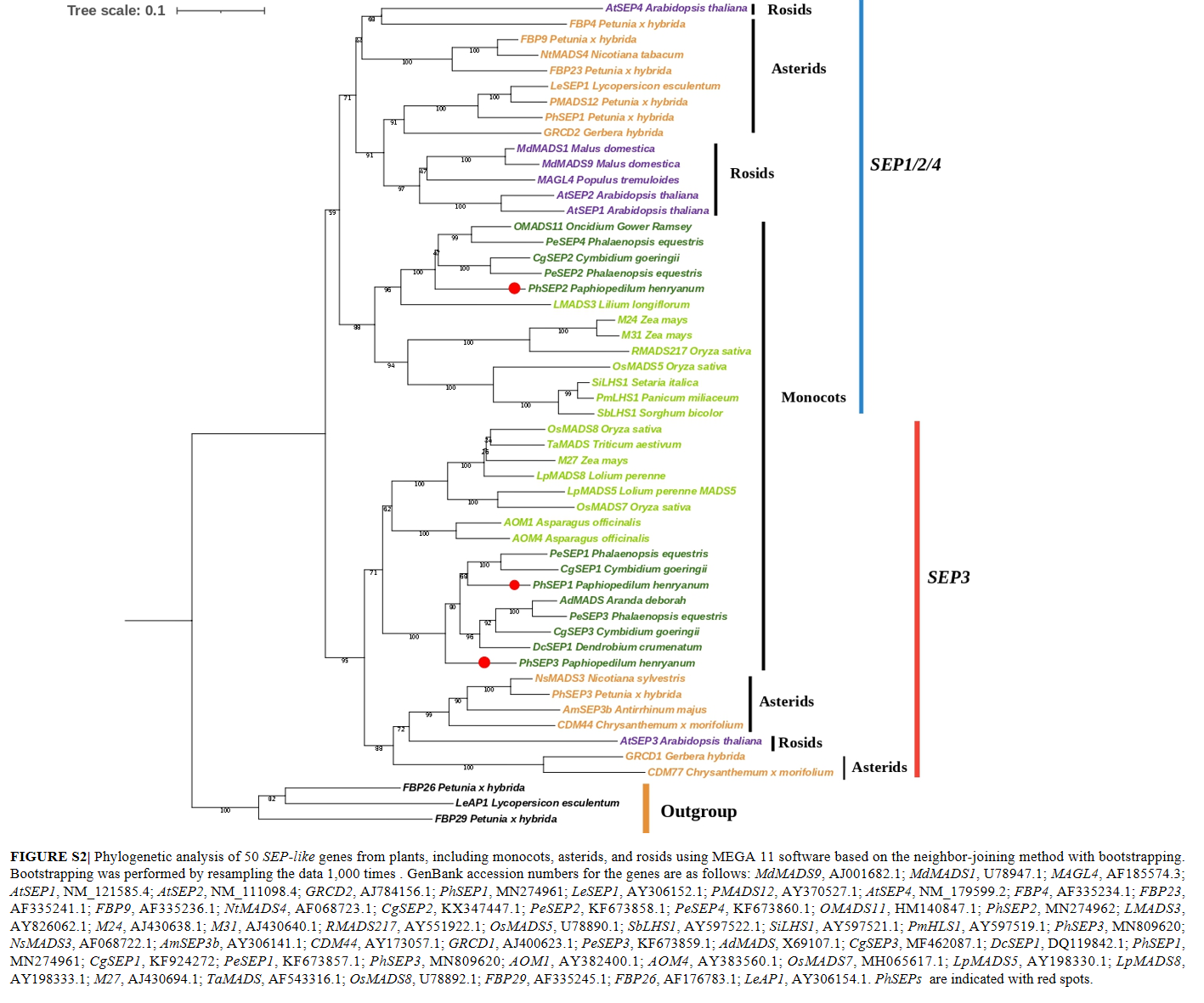

Supplement: Supplementary file 4 [file Image_2.tif]
